# *Ganoderma lucidum* extract (GLE) impairs breast cancer stem cells by targeting the STAT3 pathway

**DOI:** 10.18632/oncotarget.26294

**Published:** 2018-11-13

**Authors:** Tiffany J. Rios-Fuller, Gabriela Ortiz-Soto, Mercedes Lacourt-Ventura, Gerónimo Maldonado-Martinez, Luis A. Cubano, Robert J. Schneider, Michelle M. Martinez-Montemayor

**Affiliations:** ^1^ Universidad Central del Caribe-School of Medicine, Bayamon, Puerto Rico, United States of America; ^2^ NYU School of Medicine, New York, New York, United States of America

**Keywords:** triple negative breast cancer, cancer stem cells, aldehyde dehydrogenase, Ganoderma lucidum extract, STAT3

## Abstract

The aggressive nature of triple negative breast cancer (TNBC) may be explained in part by the presence of breast cancer stem cells (BCSCs), a subpopulation of cells, which are involved in tumor initiation, progression, metastasis, recurrence, and therapy resistance. The signal transducer and activator of transcription 3 (STAT3) pathway participates in the development and progression of BCSCs, but its role in TNBC remains unclear. Here, we report that *Ganoderma lucidum* extract (GLE), a medicinal mushroom with anticancer activity, acts on BCSCs *in vitro* and in TNBC pre-clinical animal tumor models by downregulating the STAT3 pathway. We show that GLE significantly reduces TNBC cell viability, and down-regulates total and phosphorylated STAT3 expression. This is consistent with the reduction of OCT4, NANOG and SOX2 expression, reduction in the BCSC population by loss of the ALDH1 and CD44^+^/CD24^–^ population, the deformation of mammospheres, and the strong reduction in animal tumor volume and tumor weight. Analysis of the BCSC compartment in tumors revealed that GLE decreases the STAT3 pathway and the expression of OCT4, NANOG, and SOX2 in BCSCs. These findings demonstrate that the anti-cancer activity of GLE targets BCSCs of TNBC through the downregulation of the STAT3 pathway.

## INTRODUCTION

Breast cancer is one of the leading causes of cancer deaths in women worldwide, estimated at one million cases diagnosed with more than 450,000 deaths annually [1, 2]. The clinically distinct aggressive subtype of breast cancer, triple negative breast cancer (TNBC) lacks estrogen receptors (ER), progesterone receptors (PR), and human epidermal growth factor 2 receptor (HER2) and is characterized by poor outcome [1–4]. TNBC accounts for 15–20% of all breast cancer cases, predominantly in younger patients (<50 years), distinguished by an increased tumor size and tumor grade, early recurrence, and a lower five-year overall survival rate compared with other breast cancer subtypes [1–5]. To date, treatment options for TNBC patients are chemotherapy and breast-conserving therapy (BCT), since no targeted therapy has shown to be effective for these patients [4–6]. One of the reasons why TNBC tumors are so aggressive may be explained by the presence of breast cancer stem cells (BCSCs) [7].

BCSCs are a small population of cells involved in tumor initiation, proliferation, progression, metastasis, and therapy resistance [8–10]. The major clinical concern with BCSCs is their resistance to chemotherapy, endocrine therapy, and radiotherapy [8]. Moreover, the first tumorigenic BCSCs were identified by the expression of the cell surface marker CD44, and absence of CD24 (CD44^+^/CD24^–^) and is associated with poor prognosis [10]. The presence of CD44 promotes tumorigenesis and metastasis in breast cancer, as does the absence of CD24 [9]. An additional BCSC marker is aldehyde dehydrogenase 1 (ALDH1), a detoxifying enzyme which is associated with BCSCs and increased malignancy, invasion, and metastasis [8, 9]. In addition, transcription factors involved in stem cell self-renewal and pluripotency, such as POU class 5 homeobox 1 (POU5F1 [also known as OCT4]), NANOG homeobox (NANOG), and SRY (sex determining region Y)-box 2 (SOX2) proteins, have been shown to be up-regulated in human breast cancer, and their overexpression is linked to cell transformation, tumorigenicity, tumor metastasis, and distant recurrence following chemotherapy [11, 12]. BCSCs can also be enhanced by growing as spheres (i.e. mammospheres) in serum-free, anchorage-independent, and growth factor-supplemented conditions [13, 14]. Normal human mammary epithelial cells have the ability to form mammospheres, which have an increased number of mammary stem cells and can form a functional mouse mammary gland *de novo* [15]. In another study, tumors with stem cell markers, CD44^+^/CD24^–^/Lin^–^ and ALDH1, grown as mammospheres showed an increased capacity for tumor initiation in xenograft models [16].

Many molecular signaling pathways contribute to the properties of BCSCs, including self-renewal, proliferation, survival, and differentiation [17]. According to the literature, the signal transducer and activator of transcription 3 (STAT3) is involved in many cellular processes such as proliferation, survival, anti-apoptosis, invasion, angiogenesis, and metastasis [8, 18]. More importantly, STAT3 has been shown to be highly involved in the development and progression of BCSCs [8, 9]. Evidence supports that BCSCs with the CD44^+^/CD24^–^ phenotype are regulated by the Janus Kinase 2 (JAK2)/STAT3 pathway when compared to other breast tumor cells [8]. Furthermore, subpopulations of breast cancer cells that are ALDH1 positive express higher levels of phosphorylated STAT3 (Tyr705) than cells that do not express this stem cell marker [19]. Studies have shown that NANOG together with OCT4 and SOX2, are key transcription factors involved in stem cell potency and self-renewal of embryonic stem cells, in which, OCT4 and SOX2 have been shown to be functionally dependent on STAT3 [20]. NANOG cooperates with STAT3 to maintain pluripotency and self-renewing cells, after down-regulation of NANOG, cell proliferation, colony formation, and migration are reduced in breast cancer cells [21, 22]. However, it is still unclear how the STAT3 pathway regulates the growth of CD44^+^/CD24^–^ and ALDH1 positive breast cancer cells in TNBC tumor models. Furthermore, the relationship and functionality between the self-renewal transcription factors NANOG, SOX2, and OCT4 with STAT3 is still ambiguous in TNBC models. Given the involvement of STAT3 in tumorigenesis, the development of novel therapeutic targets against STAT3 becomes a potential opportunity to prevent human malignancies, specifically TNBC.

We have been investigating the novel role of *Ganoderma lucidum* extract (GLE), also known as Reishi, a medicinal mushroom known for hundreds of years to display anti-cancer activities that has recently shown anti-tumor response and survival in cancer patients in combination with traditional chemotherapy [23]. The anticancer activity of GLE was found previously to reduce cell adhesion, proliferation, survival, and invasion, but without understanding its molecular mechanism [24–26]. GLE significantly decreases TNBC tumor volume in preclinical mouse models [27]. Finally, GLE has also been shown to induce cell cycle arrest and apoptosis in human breast cancer cells [28]. Here we provide the first evidence of a molecular mechanism for GLE anti-tumor action, demonstrating that it inhibits BCSCs by inhibiting the JAK2/STAT3 pathway and BCSC survival signaling.

## RESULTS AND DISCUSSION

### GLE decreases cell viability in TNBC cell lines

Various oncogenic signaling pathways have been investigated to identify GLE’s mechanism of action, including the AKT, MAPK/ERK, mTOR and apoptosis signaling pathways, among others [27, 29–35]. However, although modulation of these pathways has been proven, none of these pathways proved to be primary targets of GLE action.

**Figure 1:**
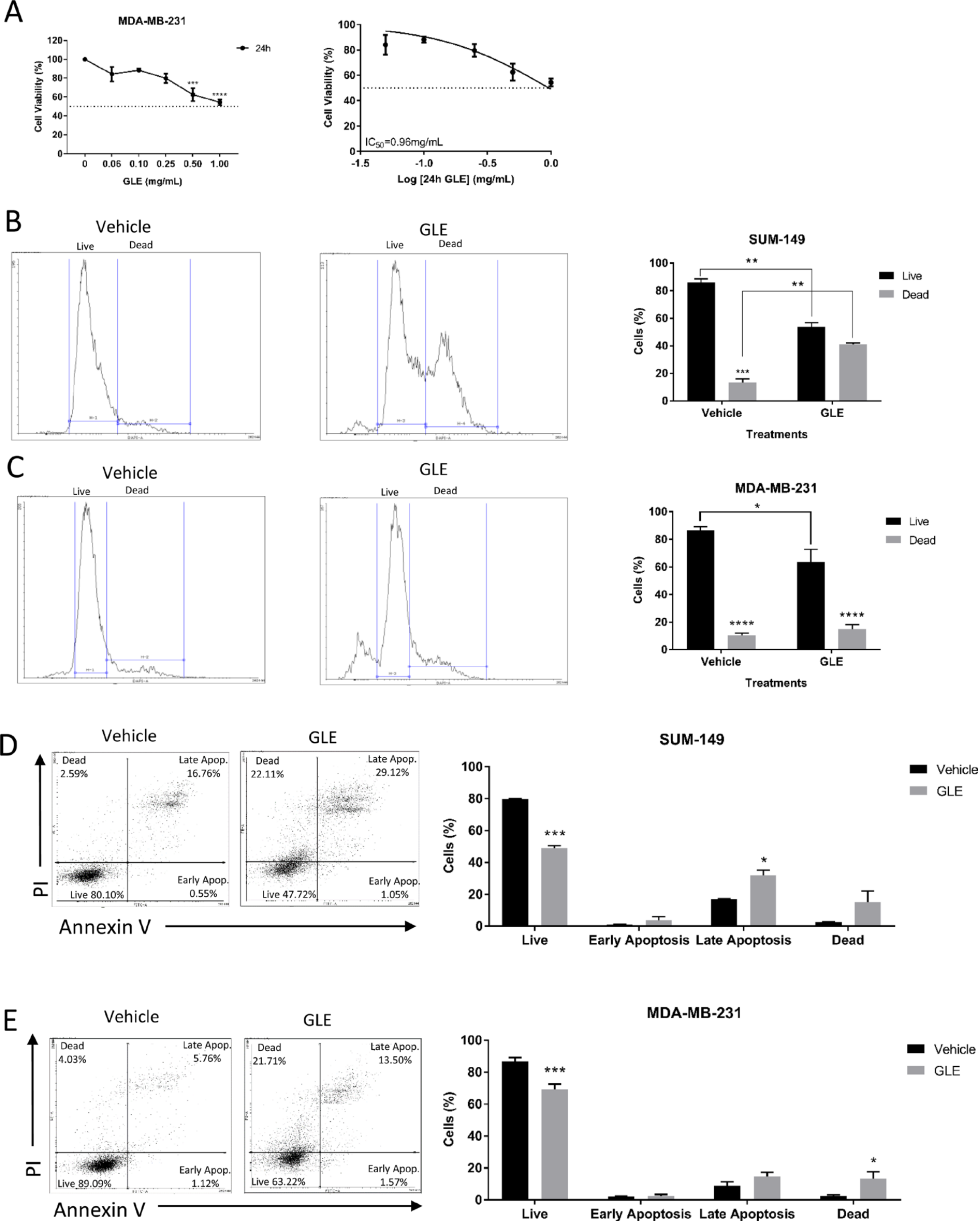
GLE decreases cell viability in TNBC cell lines (**A**) GLE significantly inhibited cell viability of MDA-MB-231 cells in a dose-dependent manner at 24 h, with a half inhibitory concentration (IC_50_) of 0.96 mg/mL. (**B**) Live/Dead Staining kit used with the SUM-149 cells revealed that GLE significantly decreased the live cells and increased the dead cells at 24 h. (**C**) While in the MDA-MB-231 cells, GLE significantly decreased the live cell population. (**D**) Annexin V/PI staining showed that GLE significantly decreased live cells and increased late apoptotic cells at 24 h in the SUM-149 cell line. (**E**) While in the MDA-MB-231 cells, GLE significantly decreases the live cells and increases the dead cells. Columns represent mean ± SEM from 2 to 3 independent experiments. ^*^P ≤ 0.05; ^**^P < 0.01; ^***^P < 0.001; ^****^P < 0.0001, was considered statistically significant compared to vehicle.

We first sought to evaluate the effects of GLE on cell viability in the triple negative breast cancer cell line, MDA-MB-231, at increasing concentrations (0.00, 0.06, 0.10, 0.25, 0.50, and 1.00 mg/mL) of GLE for 24 h. GLE significantly decreased cell viability in a dose-dependent manner by 24 h, with statistically significant reductions initiating at 0.50 mg/mL. The median inhibitory GLE concentration [IC_50_] at 24 h for MDA-MB-231 cells is 0.96 mg/mL (Figure 1A), which is consistent with previous reports demonstrating reduced sensitivity compared to other breast cancer cell lines [31, 36, 37]. The GLE IC_50_ in SUM-149 cells, another triple negative breast cancer cell line, at 24 h is 0.50 mg/mL [29]. Importantly, immortalized but not transformed MCF-10A mammary epithelial cells were unaffected at the same time-point and concentration used in these cancer cells [29]. The effect on cell proliferation and viability were quantified for both SUM-149 and MDA-MB-231 cells by flow cytometry, treated with 0.1% DMSO as a vehicle control or at their respective GLE IC_50_ concentrations of 0.50 mg/mL and 0.96 mg/mL, respectively. GLE significantly decreased the live SUM-149 cell population by 54% and increased the dead cell population by 42%, in comparison with the vehicle treatment (Figure 1B). In MDA-MB-231 cells there was a significant decrease in the live cell population in GLE treated cells when compared to vehicle (Figure 1C). Furthermore, apoptosis is increased in breast tumors with proteins that may promote or inhibit this mechanism such as Survivin, which inhibits caspases and blocks cell death, the activation of caspase 3 that cleaves regulatory proteins essential for cell survival and maintenance, and cleavage of poly(ADP-ribose) polymerase (PARP) involved in DNA repair and programmed cell death [38, 39]. Caspase 3 plays a central role in the induction of apoptosis and is responsible for the cleavage of PARP during cell death [39]. Annexin V staining assay by flow cytometry analysis confirmed that GLE significantly decreased the SUM-149 live cell population (30.5%) and increased cells in late apoptosis (15.2%) in the SUM-149 cells compared to vehicle (Figure 1D). Our results regarding GLE inducing apoptosis in the SUM-149 cells are confirmed by our immunoblot assays, in which cleaved caspase 3 and cleaved PARP expression are significantly increased in comparison to vehicle (Supplementary Figure 1A and 1B). Our results are also consistent with previous studies, in which GLE induced apoptosis in SUM-149 and other cancer cells [29, 33–35]. In MDA-MB-231 cells, GLE significantly reduced the live cell population (17.4%) and significantly increased the dead cell population (10.9%) when compared to vehicle (Figure 1E). In addition, we examined by Western Blot analysis the expression of Survivin, a protein related to apoptosis, in which GLE significantly decreased the expression compared to vehicle (Supplementary Figure 1C and 1D). Moreover, GLE significantly decreases the expression of Cyclin B1 in MDA-MB-231 cells compared to vehicle (Supplementary Figure 1C and 1D). Cyclin B1, is a protein involved in the transition from the G2 phase to mitosis, and has been found to be overexpressed in many human breast tumors being essential for survival and proliferation [40]. Our results suggest that GLE is triggering cell death by apoptosis or necrosis depending on the cell line at this time point. This is consistent with reports using additional experimental analysis to detect apoptosis, such as Hoechst Staining and DNA fragment assay, which showed that an ethanol-soluble and acidic component (ESAC) prepared from *Ganoderma lucidum* exerted antiproliferative effects by inducing apoptosis in breast cancer cell lines, including MDA-MB-231 cells [34]. Finally, washout experiments from our laboratory in both cell lines demonstrate that GLE exerts a long-term anti-cancer cell proliferation effect in cancer cell lines when cells did not recover from GLE treatment [32]. Taken together these results demonstrate that GLE significantly decreases cancer cell viability in both TNBC cells lines.

### GLE targets STAT3 and transcription factors involved in stemness

**Figure 2:**
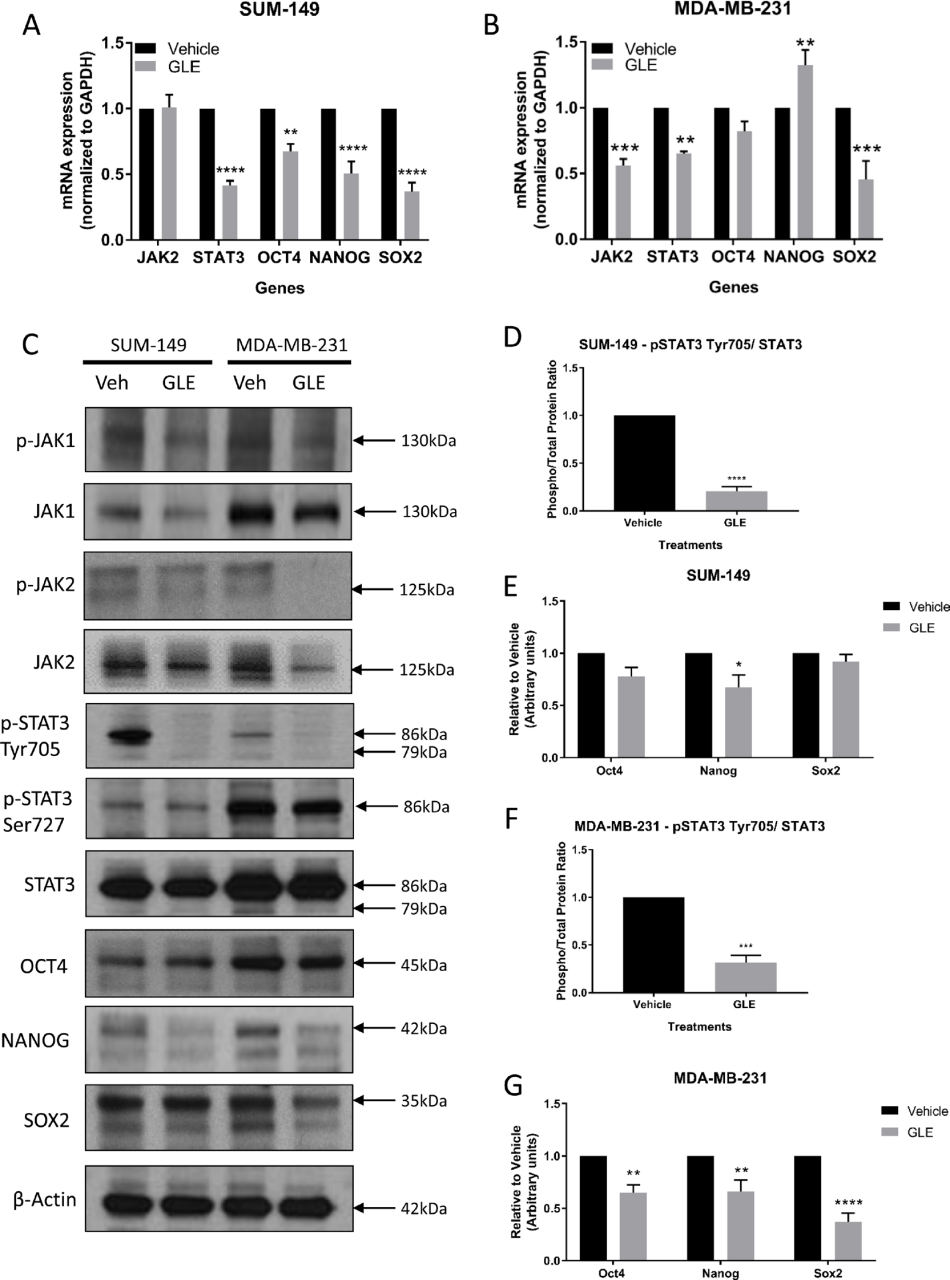
GLE targets STAT3 and transcription factors involved in cancer cell stemness mRNA expression was measured by qRT-PCR analysis and results were normalized to GAPDH. (**A**) GLE significantly decreases the gene expression of STAT3, OCT4, NANOG, and SOX2, when SUM-149 cells were treated with GLE at 24 h. (**B**) The same analyses were carried out for MDA-MB-231 cells at 24 h. Results show that GLE significantly decreases the gene expression of JAK2, STAT3, and SOX2, while increasing the expression of NANOG. (**C**) Western blot analyses of the selected proteins were analyzed, with results showing (**D**) GLE significantly decreasing the phosphorylation of STAT3 at Tyr705 and (**E**) the expression of NANOG in the SUM-149 cells at 24 h in comparison to vehicle. While in MDA-MB-231 cells, (**F**) GLE significantly decreases the phosphorylation of STAT3 at Tyr705, and (**G**) the expression of OCT4, NANOG, and SOX2 in MDA-MB-231 cells compared to vehicle. β-actin was used as a loading control. Columns represent mean ± SEM from 3 independent experiments. ^*^P ≤ 0.05; ^**^P < 0.01; ^***^P < 0.001; ^****^P < 0.0001, was considered statistically significant compared to vehicle.

STATs are a large family of transcription factors with the ability to provide instructions that mediate essential chemical signaling pathways that control fundamental functions, such as proliferation, differentiation, angiogenesis, cell death, and immune responses [41]. Among the family of STAT proteins, the transcription factor STAT3 mediates extracellular signaling through the interaction of various ligands with their respective receptors, including Interleukin-6 (IL-6), the most well-defined activator of STAT3 [42, 43]. Briefly, the binding of the cytokine IL-6 to its receptor induces receptor dimerization and assembling of JAK proteins, specifically JAK1 and JAK2, to the cytoplasmic domain of the receptor, initiating phosphorylation and activation of the JAK proteins [44]. Then, the activated tyrosine kinase JAK promotes recruitment of STAT3, once JAK and STAT3 are associated, STAT3 becomes phosphorylated on Tyr705 resulting in homodimerization and translocation to the nucleus to initiate transcription of target genes [41]. Interestingly, the JAK2 gene has been found to be amplified in TNBC tumors, and clinical and preclinical studies have shown that the JAK2/STAT3 pathway is constitutively activated in the majority of breast cancer cases, including TNBC [45–49]. STAT3 has been reported to have an essential role in maintaining the expression of genes that are important for stem cell phenotype and used as markers of CSCs [50]. More importantly, years of investigation and a recent article have revealed that the transcription factor NANOG, together with OCT4 and SOX2, play an important role in the development of malignant phenotype cells, and this may be regulated by the activation of STAT3 [51]. Given the significant inhibition of GLE on tumorigenesis and the role of CSCs in this regard, we determined the effect of GLE on the expression and activity of JAK/STAT3 signaling and the transcription factors involved in self-renewal, including OCT4, NANOG and SOX2 [52]. We first sought to determine the effect of GLE in the expression of STAT3 in both our TNBC cell lines. qRT-PCR analysis showed that GLE reduced STAT3 gene mRNA abundance by greater than 50% in SUM-149 (Figure 2A), and by 35% in MDA-MB-231 cells (Figure 2B). In our immunoblot results, we observe a decrease in phosphorylation of STAT3 at Tyr705 in both TNBC cell lines, with no change in total expression of STAT3 or the phosphorylation of STAT3 at Ser727 (Figure 2C). We show that GLE significantly decreases the phospho/total protein ratio of p-STAT3 Tyr 705 and total STAT3 in both TNBC cell lines (Figure 2D and 2F). Because STAT3 can be activated by JAK1 or JAK2, we studied the effect of GLE upstream of STAT3. In our qRT-PCR results, GLE did not reduce JAK2 gene expression in SUM-149 cells, but did decrease the gene expression in MDA-MB-231 cells (Figure 2A and 2B). However, in our immunoblot assays, GLE slightly decreases the expression of JAK1 in SUM-149 cells, and notably decreases the total expression and activation of JAK2 in MDA-MB-231 cells (Figure 2C). This indicates that GLE might be affecting STAT3 by JAK1 in the SUM-149 cells and JAK2 in the MDA-MB-231 cells. Our data is supported since the SUM-149 cells are established from primary inflammatory breast cancer (IBC) tumors, and other studies showed that JAK1 and STAT3 are activated in IBC cells compared to non-IBC cell lines [53]. We continued to measure the expression of the three transcription factors involved in self-renewal and that can be regulated by STAT3. In Figure 2A, GLE significantly decreases the mRNA expression of OCT4, NANOG, and SOX2 in SUM-149 cells. In MDA-MB-231 cells, GLE also reduced the mRNA expression of SOX2, but slightly increased expression of NANOG (Figure 2B). We also performed western blot analysis of the three proteins in both TNBC cell lines (Figure 2C). GLE significantly decreased the expression of NANOG in SUM-149 cells compared to vehicle (Figure 2E). Moreover, a significant decrease was obtained in the expression of OCT4, NANOG and SOX2 in the MDA-MB-231 cell line at 24 h in comparison with vehicle (Figure 2C and 2G). GLE, therefore, decreases the expression and activating phosphorylation of STAT3 at Tyr705, which might mediate a decreased expression of the transcription factors involved in self-renewal.

Since the JAK2/STAT3 pathway can be activated by IL-6, we measured IL-6 secretion after treatment with GLE for 24 h. GLE significantly decreased the endogenous secretion of baseline IL-6 in the MDA-MB-231 cells but not in SUM-149 cells (Supplementary Figure 1E and 1F). GLE can, therefore, regulate the IL-6/JAK2/STAT3 pathway in MDA-MB-231 cells, while downregulation of STAT3 in SUM-149 cells occurs by another upstream pathway, which still needs to be investigated (e.g. c-MET, gp130, FGFR, VEGFR, IGFR, or GPCR) [18, 54–56]. GLE inhibition of the IL-6/JAK2/STAT3 pathway in MDA-MB-231 cells confirms previous findings demonstrating the importance of this pathway in the growth of breast cancer cells [47, 57]. Furthermore, experiments were carried out to investigate the effect of GLE on the JAK2/STAT3 pathway in the MCF-10A cell line. GLE did not alter the expression of JAK2 or STAT3 in these cells (Supplementary Figure 2A and 2B). Moreover, GLE did not affect the protein expression of OCT4, NANOG or SOX2 transcription factors in MCF-10A cells (Supplementary Figure 2A and 2C). Thus, GLE does not reduce the stem cell properties in transformed but not immortalized breast epithelial cells.

### GLE reduces ALDH1 activity, the CD44^+^/CD24^–^ population and deforms mammospheres in TNBC cells

**Figure 3:**
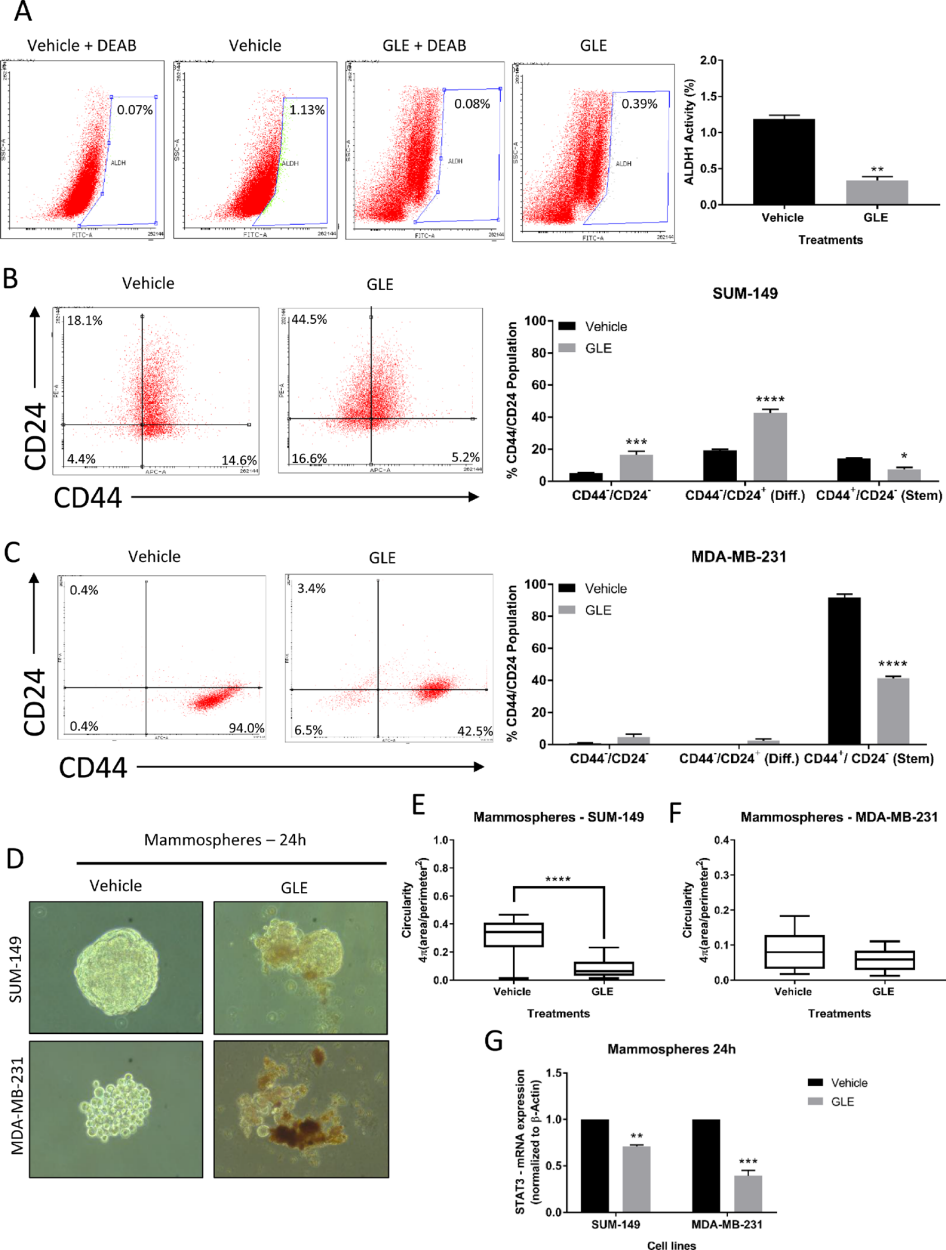
GLE reduces ALDH1 activity, CD44^+^/CD24^–^ population, and deforms mammospheres in TNBC cells (**A**) FACS images and quantified results of ALDH1 activity in MDA-MB-231 cells at 24 h reveal that GLE significantly decreases ALDH1 activity, compared to vehicle. (**B**) Representative flow cytometry dot plots of CD44 and CD24 expression in SUM-149 cells treated with GLE at 24 h show a significant increase in double-negative stained cells and a decrease in the CD44^+^/CD24^–^ (stem cell) population. (**C**) The statistical analyses performed in the MDA-MB-231 cells display that GLE significantly decreases the CD44^+^/CD24^–^ population at 24 h in comparison to vehicle. (**D**) TNBC cell lines were seeded to form mammospheres in non-serum non-adherent culture conditions in the absence (Vehicle) or presence of GLE, with results showing a decrease in size and deformation of the mammospheres at 24 h. (**E**) Quantification of the circularity for SUM-149 mammospheres shows that GLE significantly decreases the circularity at 24 h compared to vehicle, (**F**) while in the MDA-MB-231 mammospheres, GLE shows a tendency (*P <* 0.07) to decrease circularity compared to vehicle. (**G**) mRNA expression of STAT3 was measured by qRT-PCR analysis after mammospheres were treated with vehicle and GLE, with results showing that GLE significantly decreases the gene expression of STAT3 in both TNBC cell lines compared to vehicle. Results were normalized to β-actin. Columns represent mean ± SEM from 2 or 3 independent experiments. Data for the circularity analysis are from *n* = 20 replicates from 3 independent experiments. ^*^P ≤ 0.05; ^**^P < 0.01; ^***^P < 0.001; ^****^P < 0.0001, was considered statistically significant compared to vehicle.

STAT3 has been shown to be highly involved in the development and progression of BCSCs and might be a chemoresistance biomarker associated with BCSCs [8, 9, 58]. BCSCs can be identified and isolated by their markers: CD44^+^/CD24^–^ and increased aldehyde dehydrogenase 1 (ALDH1) activity [9]. In addition, a STAT3 phosphorylation inhibitor (LLL12) reduced the ALDH positive population by apoptosis induction, as well as BCSC-like cell viability, tumorsphere-forming capacity and tumor growth from ALDH^+^ breast cancer cells [19]. We, therefore, examined the effect of GLE inhibition of STAT3 on BCSC properties, including targeting the ALDH1 positive population. GLE significantly decreased ALDH1 activity from 1.13% to 0.39% (Figure 3A), almost a 3-fold reduction. Next, we measured the percent population of cells expressing CD44 and CD24 in both TNBC cell lines. We found that the CD44^+^/CD24^–^ (stem) population of SUM-149 cells was reduced from 14.6% to 5.2%, and in MDA-MB-231 cells from 94.0% to 42.5% (Figure 3B and 3C). Accordingly, GLE increased the CD44^-^/CD24^–^ population from a 4.4% to 16.6% in SUM-149 cells, and from 0.4% to 6.5% in MDA-MB-231 cells. The same experiments were performed in MCF-10A cells, in which GLE did not affect the CD44^+^/CD24^–^ (stem) population in comparison to vehicle (Supplementary Figure 2D). We continued to investigate the effect of GLE in mammosphere formation in SUM-149 and MDA-MB-231 cells after 24 h treatment with vehicle and GLE. In Figure 3D, we assessed mammosphere disruption and observed a marked decrease in the sphere size and disintegration in both TNBC cell lines. Moreover, we quantified the circularity of mammospheres in GLE treated TNBC cells. Roundness is a measure of the deviation of the ability of mammospheres to form a perfect circle where lower values indicate less circularity [59–61]. Our results show that GLE significantly decreases circularity of SUM-149 mammospheres (Figure 3E), whereas there was a tendency to a significant decrease in the MDA-MB-231 mammosphere circularity in GLE treated cells compared to vehicle (Figure 3F). According to the literature, mammospheres from different origins (i.e. cell lines or tissues) show a different morphology, where MDA-MB-231 cells displays lower mammosphere formation efficiency (MEF) compared to other breast cancer cell lines [62]. We next investigated the mRNA expression of STAT3 in mammospheres by quantitative RT-PCR. GLE treatment decreased STAT3 mRNA expression by 30% in SUM-149 cells, and by 61% in MDA-MB-231 cells when compared with vehicle (Figure 3G). These data suggest that down-regulation of STAT3 by GLE selectively targets the BCSC population in TNBC cells.

### GLE decreases CD44^+^/CD24^–^ tumor growth and inhibits the STAT3 signaling pathway *in vivo*

**Figure 4:**
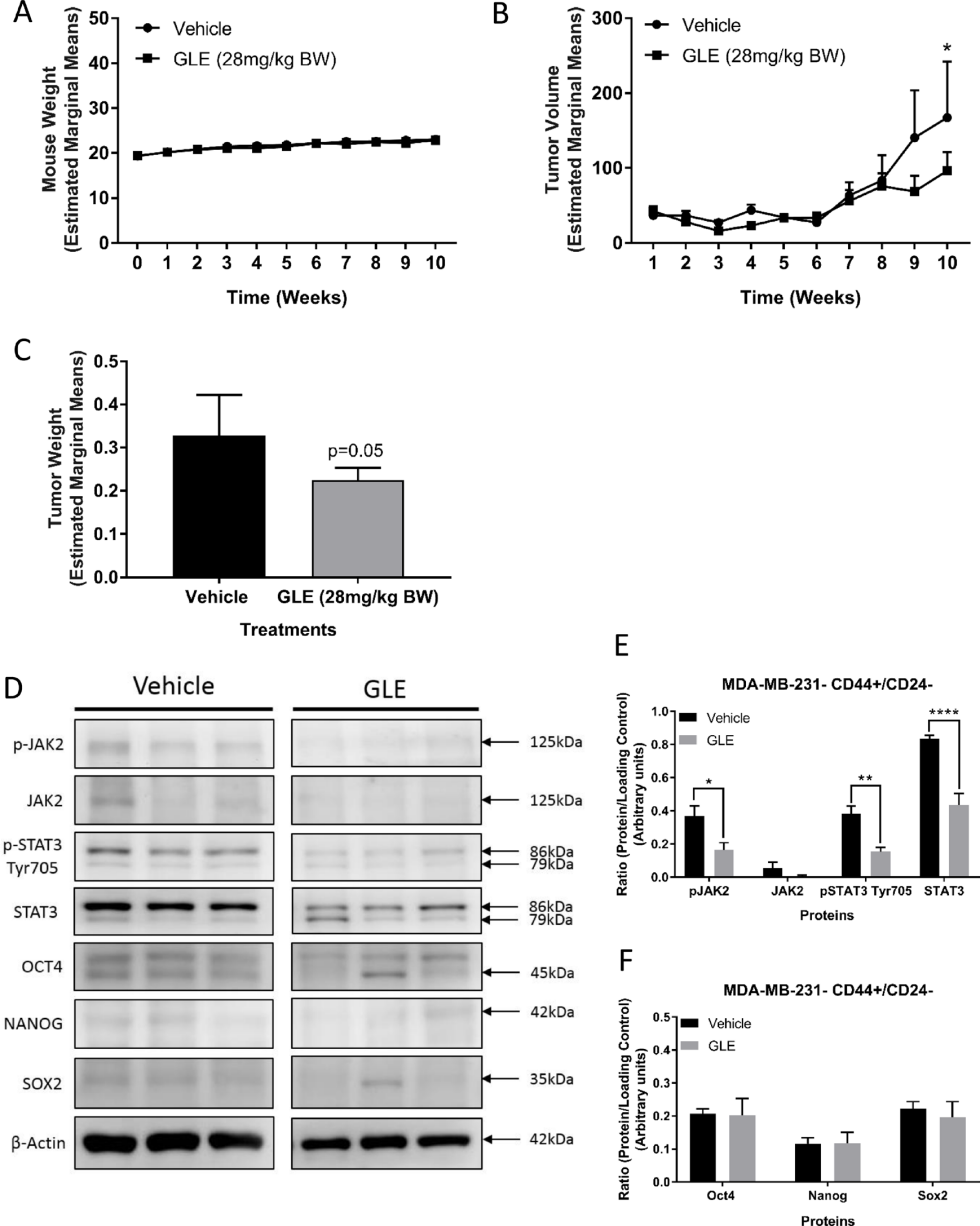
GLE decreases CD44^+^/CD24^–^ tumor growth and inhibits the STAT3 signaling pathway *in vivo* (**A**) Average mouse weights of each group. GLE treated group and vehicle treated group showed no significant differences in mouse weight. (**B**) The graph represents average tumor volumes of each group over time (10 weeks). The average tumor volume from GLE treated mice significantly decreased in comparison to the vehicle treated group. Bar represents tumor volume mean ± SEM (*n* = 7 mice, *P <* 0.05). (**C**) Average tumor weights of each group. The average tumor weight from GLE treated group was significant in comparison to the vehicle treated group (mean ± SEM, *n* = 7, *p* = 0.05). (**D**) Western blot assays of GLE effects in BCSCs tumors. (**E**) GLE decreases phospho-JAK2 (Tyr1007/1008), phospho-STAT3 (Tyr705), and total STAT3. (**F**) GLE does not change the expression of the three transcription factors (OCT4, NANOG, and SOX2) in BCSCs tumors. Total proteins were extracted from tumor tissues. Immunoblots were performed on three independent experiments with indicated antibodies. β-actin was used as a loading control. Columns represent mean ± SEM from 3 independent experiments. ^*^P ≤ 0.05; ^**^P < 0.01; ^***^P < 0.001; ^****^P < 0.0001, was considered statistically significant compared to vehicle.

As previously described, the STAT3 pathway plays a crucial role in the maintenance of BCSCs, as does the IL-6/JAK2/STAT3 pathway and increased expression of ALDH1 [8, 9, 63]. Inhibition of STAT3 also suppressed tumor growth and reduced the ALDH positive breast cancer stem cell population in mouse xenograft models [15]. As we have shown in this study and previously, MDA-MB-231 cells harbor more than 90% of CD44^+^/CD24^**–**^ population and have a higher STAT3 activation than other breast cancer cell lines [64, 65]. We, therefore, examined the tumor therapeutic potential of GLE on STAT3 signaling and BCSC stemness and viability in MDA-MB-231 cells. We found that GLE does not alter mouse weight compared to vehicle, indicating a lack of systemic toxicity (Figure 4A). Importantly, GLE significantly suppressed tumor volume (*P* < 0.05) and tumor weight (*P* = 0.05) in mice injected with sorted CD44^+^/CD24^**–**^ breast cancer stem-like cells (Figure 4B and 4C). Immunoblot analysis revealed a significant decrease in the phosphorylation of JAK2 at Tyr1007/1008 in tumors of mice treated with GLE, as well as in the expression of total STAT3 and phosphorylation of STAT3 at Tyr705 (Figure 4D and 4E). Finally, we measured the expression of all three transcription factors. In our results, there were no significant changes in the expression of all three stemness transcription factors (OCT4, NANOG, and SOX2) compared to vehicle alone (Figure 4D and 4F). These data contrast with *in vitro* results and might be a result of the interaction of the cancer stem cells with the tumor microenvironment, which has been shown to induce plasticity of cancer stem cells in cancer models [66]. As we showed in our *in vitro* results, GLE is targeting the BCSCs in tumors through downregulation of STAT3 and significantly decreases tumor volume and weight of BCSCs of the TNBC cell line, MDA-MB-231.

In summary, we observed that GLE is impairing the cell viability of TNBC cells by programmed cell death. GLE is significantly suppressing multiple properties of BCSCs such as decreasing the ALDH1 population and the CD44^+^/CD24^**–**^ stem-like population, inducing mammosphere deformation and decreasing circularity, and blocking self-renewal transcription factors expression through inhibition of STAT3 signaling. Also, GLE significantly decreased tumor volume and weight from mice injected with sorted (i.e. CD44^+^/CD24^–^) breast cancer stem cells, and significantly decreased the activation of STAT3 in these xenograft’s tumors. These results suggest that GLE may be a potential novel treatment for TNBC by targeting the BCSCs population *in vitro* and *in vivo*.

## MATERIALS AND METHODS

### Cell culture

Three breast cancer cell lines, MDA-MB-231 (ATCC^®^ HTB-26™), SUM-149 (Asterand Bioscience, Detroit, MI, USA), and MCF-10A (ATCC^®^ CRL-10317^™^) characterized for their negativity for ER, PR, HER2 molecular classification were used [2]. MDA-MB-231 cells were obtained from the American Type Culture Collection (ATCC, Manassas, VA, USA) and grown in Dulbecco’s modified Eagle’s medium (DMEM, Corning, Corning, NY, USA) containing 10% fetal bovine serum (FBS). The SUM-149 cells were obtained from Dr. Steven Ethier, Medical University of South Carolina (Charleston, SC), and grown in RPMI-1640 (Gibco - Thermo Fisher Scientific, Waltham, MA, USA) with 5% FBS [27]. Human mammary epithelial MCF-10A cells were obtained from ATCC (Manassas, VA, USA) and were grown in DMEM and Ham’s F12 medium with 10% horse serum (Millipore Sigma, St. Louis, MO, USA). All cells lines were incubated at 37° C in an atmosphere of 5% CO_2_ [29]. All cell lines were tested regularly to ensure they were free from mycoplasma infection using the Mycoplasma Detection Kit (ASB-1310001, Nordic BioSite AB, Sweden). In addition, both triple negative breast cancer cell lines were genotyped for authenticity using the Short tandem repeat (STR) profile and interspecies contamination testing services from IDEXX BioResearch (Columbia, MO, USA).

### *Ganoderma lucidum* extract

Whole mushroom *Ganoderma lucidum* extract (GLE) was purchased from Pharmanex^®^ (Provo, UT). This commercially available mushroom contains 13.5% polysaccharides, 6% triterpenes, and 1% cracked spores as described by us [27, 29]. The extract is available in capsules, where the contents were prepared at a working stock of 100 mg/mL, dissolved in 10% sterile Dimethyl Sulfoxide (DMSO) diluted (1:10, v/v) with media. The product and effective concentrations for the cells have been chosen after a literature review, and after performing dose-response curves [29]. We conduct characterization studies with FTIR, GC/MS, Cyclic Voltammetry, to ensure and assess product stability, batch-to-batch characterization, and batch-to-batch variability.

### Cell viability assay

Cells (6 × 10^4^ –1.5 × 10^5^ cells/well) were seeded and cultured for 24 h. Then, the cells were treated in duplicates with 2-fold serial dilutions of GLE for 24 h. Cells were fixed in cold 100% methanol, and nuclei stained [0.4% propidium iodide (PI)] (Millipore Sigma), and measured using a GloMax^®^ Microplate Reader (Promega, Madison, WI, USA) [29]. Cell viability was calculated as the percent of surviving cells after treatment relative to vehicle (0.1% DMSO) wells.

### Live and dead assay

Cells were analyzed using the LIVE/DEAD^™^ Fixable Dead Cell Stain Kits (#L23105, Invitrogen - Thermo Fisher Scientific) according to the manufacturer’s protocol. Cells (5 × 10^5^ cells/plate) were seeded for 48 h, and then treated with vehicle or [0.5 mg/mL for SUM-149 or 0.96 mg/mL for MDA-MB-231] of GLE for 24 h. Then harvested and rinsed in 1 mL of phosphate buffer saline (PBS), followed by resuspension of 1 × 10^6^ cells/mL in PBS. Cells were incubated with 1 µL of the reconstituted fluorescent reactive dye at room temperature for 30 min in the dark. The samples were analyzed in a Becton Dickinson BD^™^ LSR II UV flow cytometry cell analyzer (Becton, Dickinson and Company, Franklin Lakes, NJ, USA).

### Immunoblot analysis

Cells (5 × 10^5^ cells/plate) were seeded for 48 h, and then treated with vehicle or [0.5 mg/mL for SUM-149 or 0.96 mg/mL for MDA-MB-231] of GLE for 24 h, then washed with PBS and solubilized in RIPA buffer (150 mM NaCl, 50 mM Tris pH 8.0, 1% NP-40) containing Halt Phosphatase Inhibitor Cocktail (Thermo Fisher Scientific) and Protease Inhibitor Tablet (Roche Diagnostics Corporation, Indianapolis, IN, USA), and incubated for 10 min on ice. Supernatants were collected after centrifugation (13,000 RPM, 4° C, 10 min) and protein concentrations were measured with a BCA protein assay kit (Thermo Fisher Scientific). Equal quantities of protein (30 µg) were subjected to sodium dodecyl sulfate-polyacrylamide gel electrophoresis (SDS-PAGE) and electrotransferred onto a PVDF membrane (Immobilon^®^-P, Millipore Sigma). The membranes were blocked with BSA 5% and incubated at 4° C with primary antibodies purchased from Cell Signaling Technology (Danvers, MA, USA). They include JAK1 (#3332), Phospho-JAK1 (Tyr1022/1023) (#3331), JAK2 (#3230), Phospho-JAK2 (Tyr1007/1008) (#3771), STAT3 (#12640), Phospho-STAT3 (Tyr705) (#9145), Phospho-STAT3 (Ser727) (#9134), OCT4 (#2750), NANOG (#3580), SOX2 (#2748), Caspase 3 (#9662), Cleaved Caspase 3 (Asp175) (#9664), PARP (#9542), Cleaved PARP (Asp214) (#5625), Survivin (#2808), and as a loading control β-Actin (#4967). All antibodies were diluted in 5% BSA. Followed by incubation with ECL-conjugated anti-rabbit or anti-mouse IgGs (1:10,000) (GE Healthcare, Chicago, IL, USA). Signal intensity was detected using an Enhanced Chemiluminescence Kit (PerkinElmer, Madrid, Spain) and X-ray film (Denville Scientific Inc., Holliston, MA, USA).

### Enzyme-linked immunosorbent assay (ELISA) of Interleukin-6 (IL-6)

Cells (5 × 10^5^ cells/plate) were seeded for 48 h, and then treated with vehicle (0.1% DMSO) or [0.5 mg/mL for SUM-149 or 0.96 mg/mL for MDA-MB-231] of GLE for 24 h. After 24 h treatment time, the supernatant was collected. The differential expression of IL-6 was determined using a Multi-Analyte ELISArray (QIAGEN, Germantown, MD, USA) following the manufacturer’s instructions. The assay was done in duplicates. A Glomax^®^ Multi Detection System (Promega) was used for the analysis.

### Real-time quantitative RT-PCR analysis of endogenous mRNAs

Total RNA was extracted from cells using Trizol reagent (Gibco - Thermo Fisher Scientific), according to the manufacturer’s instructions. RNA concentration and purity were quantified using a NanoDrop (NanoDrop Technologies, Wilmington, DE, USA). RNA purity was obtained by the spectral parameter and by the analysis of 260/280 and 260/230 ratio. First-strand cDNA was synthesized from 1 μg of total RNA using the High-Capacity cDNA Reverse Transcription Kit (Applied Biosystems-Thermo Fisher Scientific) following the manufacturer’s instructions. Real-time quantitative PCR (qPCR) analysis was performed using 500 ng cDNA, 500 nM primers (Table 1), and Maxima SYBR Green qPCR Master Mix (2×) (Bio-Rad Laboratories, Hercules, CA, USA). The samples were analyzed with an Applied Biosystems^®^ 7500 Real-Time PCR Systems (Thermo Fisher Scientific).

**Table 1:** List of primers for real-time qPCR analyses

Gene	NCBI RefSeq	Primer sequence (F):	Primer sequence (R):	
***Janus Kinase 2 (JAK2)***	NM_004972.3	TAG ATG AGT CAA CCA GGC ATA ATG	CCG CCA CTG AGC AAA GAG	
***Signal Transducer and Activator Of Transcription 3 (STAT3)***	NM_139276.2	AGC AGC TTG ACA CAC GGT A	AAA CAC CAA AGT GGC ATG TGA	
***POU Class 5 Homeobox 1 (POU5F1, also known as OCT4)***	NM_002701.4	AGC AAA ACC CGG AGG AGT	CCA CAT CGG CCT GTG TAT ATC	
***NANOG Homeobox (NANOG)***	NM_024865.2	ATG CCT CAC ACG GAG ACT GT	AGG GCT GTC CTG AAT AAG CA	
***SRY-Box 2 (SOX2)***	NM_003106.3	GCC GAG TGG AAA CTT TTG TCG	GCA GCG TGT ACT TAT CCT TCT T	
***Actin Beta (β-Actin)***	NM_001101.4	CTT CCT TCC TGG GCA TG	GTC TTT GCG GAT GTC CAC	
***Glyceraldehyde-3-Phosphate Dehydrogenase (GAPDH)***	NM_002046.3	ATG GGG AAG GTG AAG GTC G		GGG GTC ATT GAT GGC AAC AAT A

Abbreviations: RefSeq, Reference Sequence; F, Forward; R, Reverse.

### Annexin V/PI assay

After 24 h treatment with vehicle (0.1% DMSO) or GLE [0.5 mg/mL for SUM-149 or 0.96 mg/mL for MDA-MB-231], cells were analyzed with a FITC-conjugated Annexin V Apoptosis Detection Kit I (#556547, Becton, Dickinson and Company, USA) according to the manufacturer’s protocol. Cells were harvested and rinsed in cold PBS, followed by resuspension of 1 × 10^6^ cells/mL in 1X Annexin Binding Buffer. 1 × 10^5^ cells were incubated with 5 µL of FITC Annexin V (51-65874X, Becton, Dickinson and Company, USA) and 5 µL of PI (51-66211E, Becton, Dickinson and Company, USA) at room temperature for 15 min in the dark. In each sample 400 µL of 1X Annexin Binding Buffer was added. The samples were analyzed with a Becton Dickinson BD^™^ LSR II UV flow cytometry cell analyzer (Becton, Dickinson and Company, USA).

### Aldefluor assay

The ALDEFLUOR™ assay kit (#01700, StemCell^™^ Technologies, Vancouver, BC) was used to assess ALDH enzymatic activity according to the manufacturer’s protocol. Briefly, after 24 h treatment, SUM-149 and MDA-MB-231 cells were incubated for 40–45 min at 37° C in ALDEFLUOR assay buffer containing the ALDH protein substrate BODIPY-aminoacetaldehyde (BAAA, 1 µM per 1 × 10^6^ cells). As a specific inhibitor of ALDH, 50 mM diethylamino-benzaldehyde (DEAB) was used as a negative control. Aldefluor stained cells were analyzed with a Becton Dickinson BD^™^ LSR II UV flow cytometry cell analyzer (Becton, Dickinson and Company, USA).

### CD44^+^/CD24^–^ staining analysis

CD44^+^/CD24^–^ staining analysis was used to identify CSC-like cell populations. After 24 h treatment, a total of 2.5 × 10^3^ cells were incubated in the dark for 35–40 min at 4° C with PE-conjugated anti-human CD24 (#555428, BD Pharmingen™) and APC-conjugated anti-human CD44 (#559942, BD Pharmingen™) antibodies. Unbound antibody was washed off and cells were analyzed by flow cytometry on a Becton Dickinson BD™ LSR II UV flow cytometry cell analyzer (Becton, Dickinson and Company, USA).

### Mammosphere formation assay

SUM-149 cells for mammosphere formation were cultured in Ham’s F-12 medium containing 20 ng/mL EGF (Sigma), 20 ng/mL basic FGF (Fisher Scientific), 1X B27 (Invitrogen), 4 ng/mL Heparin (Sigma), 5 µg/mL insulin, 1 µg/mL hydrocortisone, 100 units/mL penicillin and 100 units/mL streptomycin. The MDA-MB-231 were cultured in HuMEC basal serum-free medium (Gibco), supplemented with B27 (1:50, Invitrogen, Carlsbad, CA, USA), 20 ng/mL basic fibroblast growth factor (bFGF, Sigma-Aldrich), 20 ng/mL human epidermal growth factor (EGF, Sigma-Aldrich), 4 μg/mL heparin, 1% antibiotic-antimycotic agent, and 15 μg/mL gentamycin. Cells were trypsinized, passed through a 40 µm cell strainer (BD Falcon), seeded in ultra-low attachment plates (Corning), cultured for 7–10 days at a density of 10,000 cells/mL for SUM-149 and 5,000 cells/mL for the MDA-MB-231 cells and the experiments were performed after the 3rd passage. The mammospheres were treated with vehicle or GLE for 24 h and analyzed using an inverted microscope. Photos were acquired with ZEN software from ZEISS Microscopy (Oberkochen, Germany). Circularity was calculated using the formula 4π (Area/Perimeter^2^) using Image J [59–61, 67].

### *In vivo* study

To test tumorigenicity we injected limiting dilutions of 5.0 × 10^3^ sorted (CD44^+^/CD24^–^) MDA-MB-231 BCSCs cells in 100 µL of Matrigel (CB-40230A, BD Biosciences, San Jose, CA, USA) solution (1:1 dilution of Matrigel with DMEM medium), into 7 mice/group to achieve adequate statistical power. The cells were injected into the mammary fat pad of 4-wk-old female severe combined immunodeficient (SCID) mice [Charles River Laboratories International (Wilmington, MA, USA)]. One-week post-inoculation, mice were randomly divided into vehicle and GLE experimental groups. The treatments began once palpable tumors were detected (∼1 wk post-inoculation). Mice were orally gavaged every day with vehicle or 28 mg/kg BW of GLE in 10% ethanol for 10 wks. Mice were housed under specific pathogen-free conditions, were given 2920X Teklad Global Rodent Diet (Harlan Laboratories, Indianapolis, IN, USA) and sterile water. Mouse weights and tumor volume (calipers measurements) were measured weekly. Tumor volume (mm^3^) was calculated: [π/6(L)(W)(H)]. Relative tumor volume was calculated as [(average tumor volume ratio on week “n”)/average tumor volume on week-1]. Mice were housed and handled in accordance with the Universidad Central del Caribe School of Medicine, Institutional Animal Care and Use Committee (UCC-IACUC), and the National Institute of Health (NIH) principles and guidelines.

### Statistical analysis

For *in vitro* experiments; normality distribution criteria was assessed via a Shapiro-Wilk test. Bivariate schema was done via Ordinary one-way ANOVA with Dunnett’s multiple comparisons post hoc test or Factorial ANOVA with Bonferroni’s correction. To account for the variance homoscedasticity, a Levene test with a Brown-Forsythe correction was performed. Mammosphere circularity quantification was performed with a non-parametric *t*-test approach using the independent samples Mann–Whitney test. The results are presented as mean ± SEM. Calculations of the IC_50_s were done with dose-response curve fittings using the non-linear regression parameter: dose-response – inhibition (log [inhibitor] vs normalized response). For gene expression studies in both TNBC cell lines, vehicle and GLE treatment were individually assessed using the 2^(–∆Ct)^ formula by comparing their relative gene expression to the expression of the reference genes. The *p-*values for gene expression PCR array analysis was calculated based on one-way ANOVA of the replicate 2^(–∆Ct)^ values for each gene in the control group and treatment groups. Statistical significance was set at *p* ≤ 0.05; excluding the normality tests. All calculations were done using GraphPad Prism v6.0 (San Diego, CA, USA).

For *in vivo* experiments, a ten week (W1–W10) statistical model with two comparison groups was done as follows: *vehicle* and *GLE 28 mg/kg.* Normality diagnostics were performed using the Shapiro-Wilk estimator. Frequencies, percentages, central tendency and dispersion measures were calculated to assess the raw distribution of the study variables. To account for the time horizon as a statistical unit, a General Linear Model Repeated Measures ANOVA (GLMRMA) approach was used in order to calculate estimated marginal means. A Mauchly’s test of sphericity was performed to assess if our models have or not the assumption of compound symmetry. If non-significant, we report the univariate results with an Epsilon correction; if significant; we report the multivariate results using the Pillai’s trace estimator. Either of the last explained factors was used to evaluate the time effect in our models. A test of between-subjects effect was applied to perceive statistical differences between the groups per block. The estimated marginal means will be reported. The significant level (α) was set to ≤0.05, excluding normality test criteria. IBM Statistical Package for Social Sciences (IBM-SPSS, Chicago, IL, USA) v.23.0 for Windows was used. To assess mouse and tumor weight in our dataset, a normality diagnostic was performed using the Shapiro-Francia estimator. Central tendency measures and dispersion measures were calculated to evaluate the raw distribution. An independent sample *t*-test was performed to compare two comparison groups as follows: *vehicle* and *GLE 28 mg/kg BW*. Variance homoscedasticity was evaluated using the Levene protocol. The significant level (α) was set to *P* ≤ 0.05; except for the normality and variance homogeneity tests. IBM Statistical Package for Social Sciences (IBM-SPSS, Chicago, IL, USA) v.23.0 for Windows was used.

## SUPPLEMENTARY MATERIALS FIGURES


