# Supplementary material for: Ganoderma lucidum extract (GLE) impairs breast cancer stem cells by targeting the STAT3 pathway
Source: Oncotarget. 2018 Nov 13;9(89):35907–21. doi: 10.18632/oncotarget.26294 (PMC6267592; doi:10.18632/oncotarget.26294)
Supplement: Supplementary file 1 [file oncotarget-09-35907-s001.pdf]

Ganoderma lucidum extract (GLE) impairs breast cancer stem cells by targeting the STAT3 pathway

SUPPLEMENTARY MATERIALS

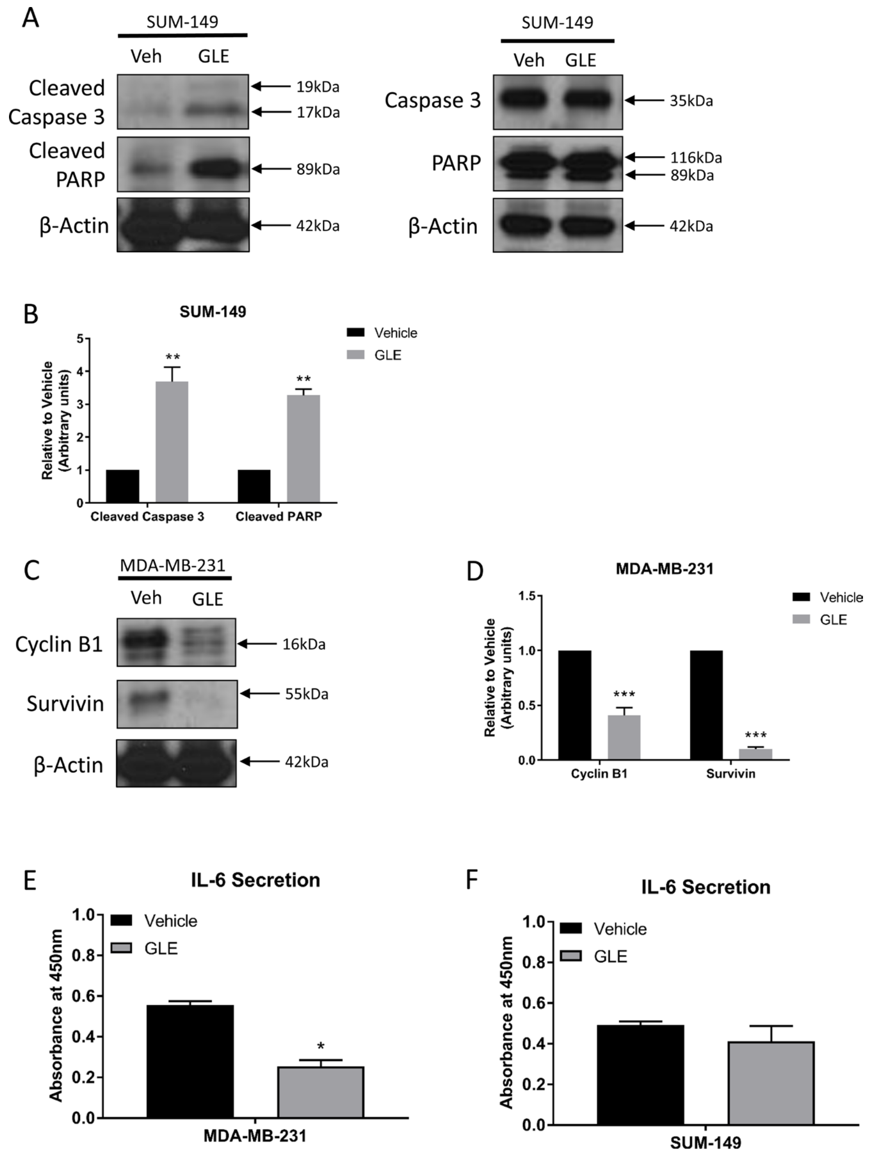

Supplementary Figure 1: GLE decreases levels of proteins involved in cell death and proliferation in both TNBC cell lines and decreases IL-6 secretion in MDA-MB-231 cells. (**A**) Western blot analyses of proteins related with apoptosis (Cleaved Caspase 3, Caspase 3, Cleaved PARP, and PARP) were analyzed, with results showing (**B**) GLE significantly increases the expression of cleaved caspase 3 and cleaved PARP in SUM-149 cells at 24 h in comparison to the vehicle. (**C**) The expression of cell proliferation proteins (Cyclin B1 and Survivin) by immunoblots assays shows that (**D**) GLE significantly decreases the expression of both proteins in MDA-MB-231 cells at 24 h in comparison to the vehicle. For both experiments, β-actin was used as a loading control. ELISA assays were performed in MDA-MB-231 cells (**E**), and SUM-149 cells (**F**), to detect IL-6 secretion after treatment with GLE for 24 h. Results show a decrease in IL-6 secretion in MDA-MB-231 cells, but not in the SUM-149 cell line. Columns represent mean ± SEM from 2 independent experiments. $^{*}P \leq 0.05$; $^{**}P < 0.01$; $^{***}P < 0.001$, was considered statistically significant compared to vehicle.

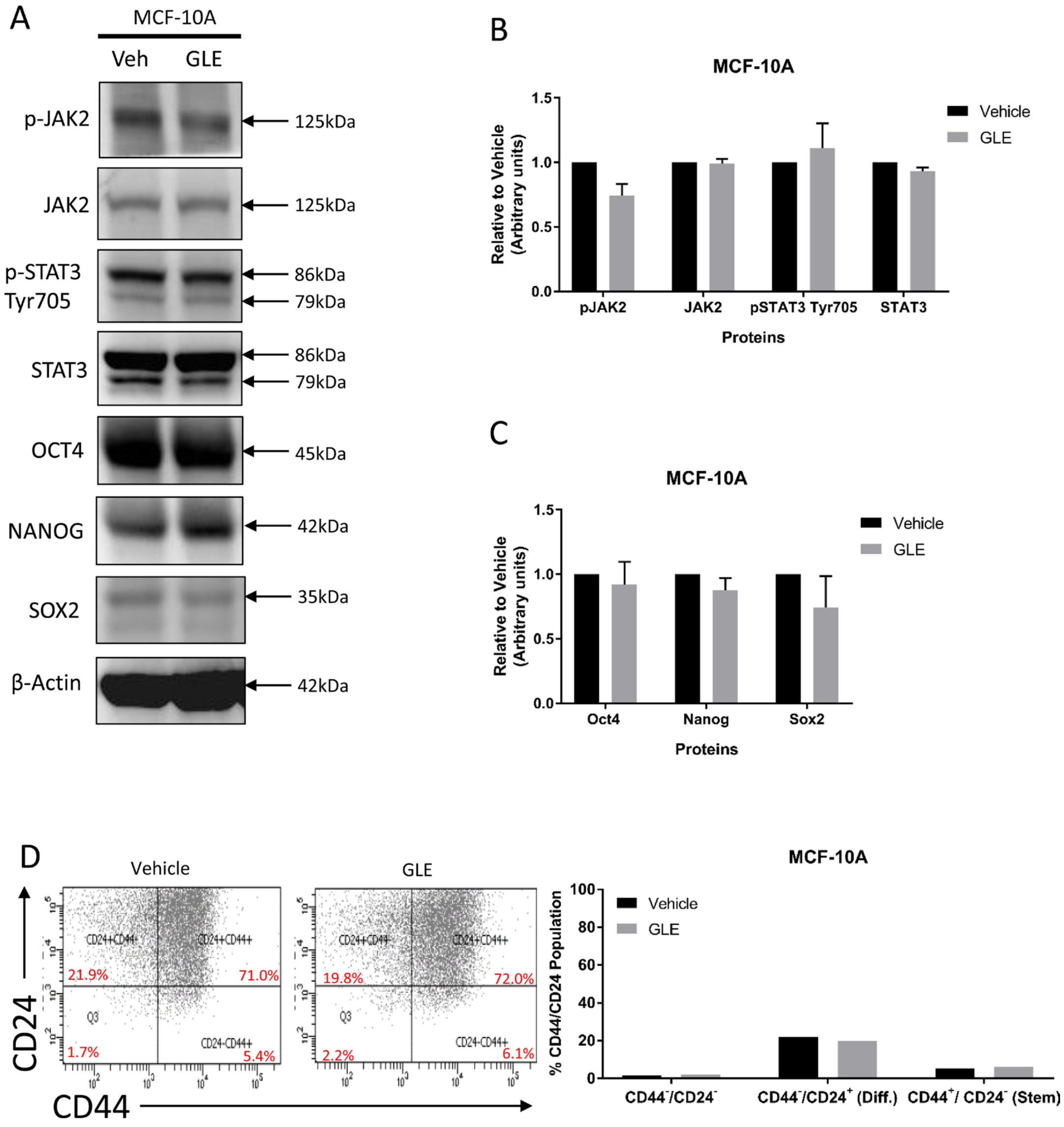

Supplementary Figure 2: GLE treatment does not decrease protein expression or the CD44^{+}/CD24^{-} population in MCF-10A cells. (**A–C**) Western blot analysis of all selected proteins was performed in MCF-10A cells at 24 h, in which, GLE does not change protein expression of selected proteins, using β-actin as a loading control. (**D**) Flow cytometry analysis to measure CD44 and CD24 expression in the MCF-10A cell line treated with vehicle and GLE at 24 h, shows that GLE does not affect the CD44^{+}/CD24^{-} (stem cell) population in non-cancerous mammary epithelial cells. The columns in the western blot analysis represent mean ± SEM from 3 independent experiments. The columns in the flow cytometry analysis represent mean ± SEM from 1 independent experiment.
